# Optimising Multispectral Active Fluorescence to Distinguish the Photosynthetic Variability of Cyanobacteria and Algae

**DOI:** 10.3390/s23010461

**Published:** 2023-01-01

**Authors:** Emilie Courtecuisse, Elias Marchetti, Kevin Oxborough, Peter D. Hunter, Evangelos Spyrakos, Gavin H. Tilstone, Stefan G. H. Simis

**Affiliations:** 1 Plymouth Marine Laboratory, Prospect Place, Plymouth PL1 3DH, UK; 2 School of Biological and Marine Sciences, University of Plymouth, Plymouth PL4 8AA, UK; 3 Chelsea Technologies Ltd., 55 Central Avenue West Molesey, Surrey KT8 2QZ, UK; 4 Faculty of Natural Sciences, University of Stirling, Stirling FK9 4LA, UK

**Keywords:** active fluorescence, multispectral, phytoplankton, cyanobacteria, algae, population dynamics, limnology

## Abstract

This study assesses the ability of a new active fluorometer, the LabSTAF, to diagnostically assess the physiology of freshwater cyanobacteria in a reservoir exhibiting annual blooms. Specifically, we analyse the correlation of relative cyanobacteria abundance with photosynthetic parameters derived from fluorescence light curves (FLCs) obtained using several combinations of excitation wavebands, photosystem II (PSII) excitation spectra and the emission ratio of 730 over 685 nm (F_o_(730/685)) using excitation protocols with varying degrees of sensitivity to cyanobacteria and algae. FLCs using blue excitation (B) and green–orange–red (GOR) excitation wavebands capture physiology parameters of algae and cyanobacteria, respectively. The green–orange (GO) protocol, expected to have the best diagnostic properties for cyanobacteria, did not guarantee PSII saturation. PSII excitation spectra showed distinct response from cyanobacteria and algae, depending on spectral optimisation of the light dose. F_o_(730/685), obtained using a combination of GOR excitation wavebands, F_o_(GOR, 730/685), showed a significant correlation with the relative abundance of cyanobacteria (linear regression, *p*-value < 0.01, adjusted R^2^ = 0.42). We recommend using, in parallel, F_o_(GOR, 730/685), PSII excitation spectra (appropriately optimised for cyanobacteria versus algae), and physiological parameters derived from the FLCs obtained with GOR and B protocols to assess the physiology of cyanobacteria and to ultimately predict their growth. Higher intensity LEDs (G and O) should be considered to reach PSII saturation to further increase diagnostic sensitivity to the cyanobacteria component of the community.

## 1. Introduction

Anthropogenic activities and climate change are primarily responsible for the increased biomass and occurrence of harmful algal blooms (HABs) [1,2,3]. In freshwater systems, eutrophic conditions promote cyanobacteria dominance [4,5,6], with the added risk of proliferation of toxin-producing species [7,8]. Although not every species of cyanobacteria that forms blooms is toxic, and not every strain of toxin-producing species will always produce toxins [9], regular monitoring for cyanobacteria blooms is critical to manage risk of toxin exposure to wildlife, recreational use or through water consumption, and to limit the economic cost of mitigation measures. Risk management may include regulation of nutrient loads into water bodies or access to them. Monitoring is required at the global scale and therefore requires cost-efficient methods. Regular monitoring should be able to assess the growth and physiology of cyanobacteria and to determine the status of the bloom or the potential for a bloom to form.

Current monitoring techniques include microscopy identification and quantification, toxin analysis [10,11,12], genetics and genomic detection [13,14], and methods based on optical characteristics such as in vivo fluorometry [15], flow cytometry [16] and imaging flow cytometry [17]. Microscopic identification and quantification, toxicity analysis and genetic methods are highly diagnostic but can be time-consuming and costly. Optical methods, other than microscopy, include diagnostics based on size spectrum analysis, morphology, and absorption and fluorescence characteristics. With the possible exception of image-based classification, which requires training and supervision, these methods are not able to discriminate taxonomic detail beyond functional groups. Fluorometry-based techniques are also limited to distinguishing variations in photosynthetic pigment composition at group-level, but fluorescence can be used to determine photophysiological traits that can be related to population growth.

Several types of fluorometers exist to discriminate between phytoplankton groups, including cyanobacteria. The most affordable fluorometer designs (mostly handheld and sometimes submersible) measure fluorescence from single or multiple excitation wavebands and generally one emission waveband [18,19]. These instruments assist in obtaining phytoplankton biomass estimates but are not able to assess phytoplankton physiology [20]. In contrast, saturating flash fluorometers measure variable fluorescence from which photochemical efficiency can be determined [18]. The most elaborate fluorometers in this category saturate photosystem II (PSII) within a single turnover of all reaction centres, while fluorometers operating with a longer ‘multiple-turnover’ flash can be more affordable [20]. From the fluorescence emission during the saturating flash, the minimum quantum yield of fluorescence F_o_ (in dark-adapted state, all reaction centres opened) and the maximum quantum yield of fluorescence F_m_ (all reaction centres closed) are obtained. From the determination of F_o_ and F_m_, the variable fluorescence F_v_ (from F_m_–F_o_) and the maximum charge separation at PSII (F_v_/F_m_) are calculated. By further modulating ambient light availability, daily photosynthetic rates can be modelled from the fluorescence response [21], which in turn can be combined with nutrient and light availability in the natural environment to model and predict the growth of phytoplankton in a specific sample. Few studies to date have attempted to separate the fluorescence response from phytoplankton associated with specific pigment groups from natural samples [22,23].

Due to overlapping photosynthetic absorption spectra, a combination of fluorescence markers is required to estimate the primary production and photochemistry efficiency of cyanobacteria. Several existing variable fluorescence instruments have multiple excitation wavebands to target different spectral groups [24,25,26]. To distinguish the fluorescence response of cyanobacteria in the community, the fluorometer requires at least an excitation waveband which excites the main light-harvesting pigments, the phycobilipigments allophycocyanin, phycoerythrin and phycocyanin [15,27,28,29]. Although rhodophytes and glaucophytes are also known to possess these pigments, their abundance is unlikely to be high in environments susceptible to cyanobacteria blooms [30,31,32]. To accurately interpret the fluorescence signal and assess the physiology of cyanobacteria, excitation protocols targeting cyanobacteria should be able to fully saturate PSII through these diagnostic pigments. Moreover, the choice of emission wavebands for fluorometers is important to consider. Fluorescence emission is recorded at PSII chlorophyll *a* (chla) emission around 685 nm in most fluorometers. In cyanobacteria, most of the chla is associated with photosystem I (PSI) rather than PSII [33] and therefore has non-variable fluorescence [34]. The interpretation of the fluorescence signal for the emission of PSII chla can be complex if the sample contains cyanobacteria. Consistently higher emission ratios of 730 over 685 nm and 660 over 685 nm in cyanobacteria cultures compared to algal cultures was shown by [35]. Consequently, they suggested implementation of an emission waveband centred on phycobilisomal emission around 660 nm and an emission waveband centred around PSI chla at 730 nm. Single-turnover fluorometers with emission at 650 nm have not been produced, possibly due to the challenge of cross-talk between excitation sources and emission filters centred on 650 nm.

A new fluorometer, the LabSTAF (Chelsea Technologies Group, UK), shows increased potential to target cyanobacteria in the natural environment. The LabSTAF is a portable robust and compact instrument suited for regular on-site monitoring. The instrument is well suited for long-term continuous and autonomous measurements due to a number of software and hardware features. These include a mixing feature with a flow-through stirrer, temperature control, a sample exchange feature using a separate peristaltic pump controlled by the instrument, as well as an optional cleaning cycle using the pump and additional solenoid valve controller. The instrument has high signal sensitivity (suitable for oligotrophic conditions), which means that the full seasonal phytoplankton variability may be observed. Moreover, the instrument introduces several optical features which increase its potential to target distinct pigment groups, including those diagnostic of cyanobacteria. These include multiple excitation wavebands (see below), emission detection using 685 nm and 730 nm bandpass filters and the automated function to measure photosynthetic excitation spectra. The suitability of these specific features to target cyanobacterial photophysiology is discussed below.

The instrument is based on the concept of a Single Turnover Active Fluorometer (STAF) and uses seven excitation wavebands centered at 416, 452, 473, 495, 534, 594 and 622 nm, which can be combined to target a range of photosynthetic pigments based on their spectral characteristics. Wavebands at 534 (green) and 594 nm (orange) are particularly useful, as these target different forms of phycoerythrin that are common in both oceanic and freshwater cyanobacteria, as well as the short-wavelength tail of phycocyanin absorption [36]. The LabSTAF is equipped with a broad-spectrum actinic light source to create Fluorescence Light Curves (FLCs) that are synonymous with photosynthesis–irradiance curves used to estimate and model primary production. The single turnover variable fluorescence is recorded at the waveband 685 nm to target PSII chla. In addition it obtains F_o_, F_m_ and F_v_ at 730 nm at the start of a measurement sequence, using a linear actuator to switch between 685 nm and 730 nm bandpass filters (± 10 nm FWHM). This Dual Waveband Measurement (DWM) configuration was initially designed to correct for the pigment packaging effect [37,38]. We propose that this configuration may also be sensitive to the presence of cyanobacteria due to allocation of chla to PSI rather than PSII, resulting in a higher 730 over 685 nm emission ratio when chla light absorption is targeted. The LabSTAF is further preconfigured to measure the photosynthetic excitation spectrum across the seven excitation wavebands. This is done using one of two distinct protocols, differing in the light dose used at the spectral excitation maximum of algal and cyanobacterial pigment groups, respectively.

In this study, we assess the ability of the LabSTAF fluorometer to target physiological properties of cyanobacteria in a freshwater reservoir where cyanobacteria form annual blooms. Various combinations of fluorescence markers are tested and compared against relative phytoplankton abundance and nutrient availability. Specifically, we analyse the correlation of relative cyanobacteria abundance with photosynthetic parameters derived from FLCs obtained using combinations of excitation wavebands, the interpretation of PSII excitation spectra (both prokaryotic cyanobacteria and eukaryotic algae-optimised protocols), and the emission ratio of 730 over 685 nm obtained with excitation protocols targeting cyanobacteria versus algae. Appropriate predictors of cyanobacterial photophysiology within the phytoplankton community are expected to reflect a positive response of cyanobacteria to conditions favouring their growth, such as reduced N:P ratios and following depletion of silicate in the natural succession from spring (diatoms abundant) to summer (cyanobacteria dominant) community composition. Moreover, we expect fluorescence dynamics to reflect the phytoplankton group abundance estimates from microscopy counts. Comparing the various options for light-excitation protocols, we hypothesise that the photophysiological characterisation of algae and cyanobacteria is best achieved by interpreting single-turnover fluorescence from blue and green–orange light to target diagnostic pigment groups. Saturation and specificity of cyanobacteria fluorescence from green–orange excitation is the most critical test of the system, due to lower excitation energy achieved with light emitting diodes and the overlap with absorption by phycobilipigments and short-wavelength tails of both chlorophyll *c* and chla in this range of the spectrum.

## 2. Materials and Methods

### 2.1. Study Site

**Figure 1:**
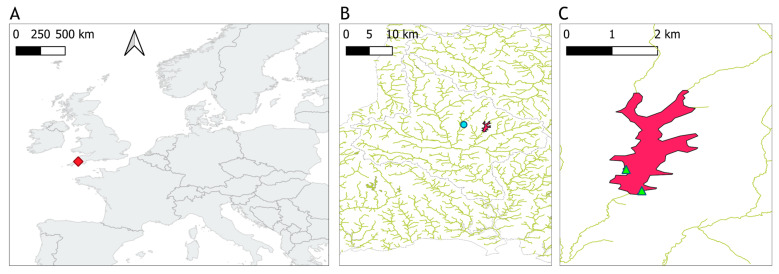
Location of Roadford Lake. (**A**) The location of the reservoir indicated with a red diamond marker. (**B**) The meteorological station “Virginstow, Beaworthy” represented by a turquoise circle to the west of the reservoir in magenta color. (**C**) Green triangles represent the sampling points on the reservoir.

Roadford Lake is the largest drinking water reservoir in Devon County in the United Kingdom (Figure 1), part of the catchment of the river Tamar. It has a net storage of 34,500 million litres and a surface area of 295 hectares. Water is pumped from a draw-off tower situated in the south part of the reservoir, near the dam where there is also an overflow tower [39]. The lake is fed by the river Wolf in the northeast and from Westweek inlet, a stream to the northwest.

**Figure 2:**
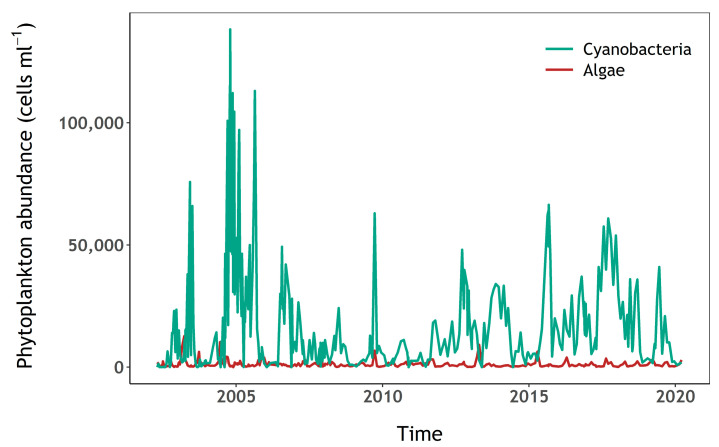
Algae and cyanobacteria abundance (cells mL^−^^1^) at Roadford Lake.

High abundances of cyanobacteria have been recorded in samples analysed by the water utility company using light microscopy, since at least spring 2002, and often year-round (Figure 2, see Section 2.3 for methods). Water samples for this study, 23 in total, were collected between 12 April and the 16 October 2020. Samples were taken every two weeks during the sampling period, and weekly during the summer, when high cyanobacteria biomass was expected. Surface water was sampled from a jetty near the western shore with a bucket in the first 0.5 m from the surface in approximately 3 m-deep water (depending on reservoir volume), except for the first seven samples which were taken directly from the east shore whilst access to the jetty was restricted (Figure 1C). During calm weather, any accumulation of cyanobacteria visible at the surface was mixed with the surface water to ensure that the sample was homogenously representative of conditions in the top layer. Water temperature was recorded at the time of sampling, and approximately 2 litres of water were brought to the laboratory in an insulated container, for further analysis on the same day. Total daily rainfall was retrieved from the National Meteorological Library and Archive of the UK Met Office for station “Virginstow, Beaworthy“ (50°42′36.0″ N 4°17′45.6″ W) which is located around 4 km west of Roadford Lake (Figure 1).

### 2.2. Single-Turnover Fluorescence

A dedicated LabSTAF instrument (serial number 19-0105-006) was used to characterise the photophysiology of phytoplankton at Roadford Lake. FLCs were measured on samples taken from 29 May to 16 October 2020, using the range of fluorescence excitation protocols described in Table 1. These protocols were selected to target semi-isolated pigment groups and the associated phytoplankton taxa through combinations of excitation wavebands expected to lead to full saturation of PSII, based on the work of [40,41]. Protocols differed in saturation pulse length (100 or 200 µs) to accommodate saturation, and the combination of excitation wavebands denoted B(lue) = 452 nm, G(reen) = 534 nm, O(range) = 594 nm, and R(ed) = 622 nm (Table 1). The Light-Emitting Diodes (LEDs) used to provide the excitation energy were always set to their maximum intensities (G = 10,500, O = 2896 and R = 7594 μmol photons m^−^^2^ s^−^^1^) except for the B LED which adapted automatically to adjust to the phytoplankton composition and abundance for the B protocol (2 are implemented in the instrument, B1 ranged between 14,108 and 16,819 μmol photons m^−^^2^ s^−^^1^ and B2 between 14,068 and 16,719 μmol photons m^−^^2^ s^−^^1^) (Table 1). Optimal B intensity values within the GORB and GOB protocols were determined prior to any measurements, from exposure using only the B LEDs with a pulse length of 200 μs. These values for the B LED were then adopted in the GORB and GOB protocols with the aim to see if results from separate protocols were additive (Table 1). Each FLC included 12 actinic light steps from 0 to 1200 μmol photons m^−^^2^ s^−^^1^. The actinic light spectra had a peak at 455 nm and shoulder from 480 nm to 655 nm. Samples were dark adapted for at least 20 min before each analysis to allow all reaction centres to open to correctly measure F_o_.

Photosynthetic parameters are expressed here as a function of excitation and emission waveband (λ_ex_, λ_em_). FLCs parameters and excitation spectrum were reported only for the emission band (λ_em_) 685 nm, but single turnover curves were also recorded at λ_em_ = 730 nm. Where multiple excitation wavebands are considered, the excitation waveband is referred to by the abbreviations given in Table 1 (e.g., B, GOR). Several photosynthetic parameters were determined from each FLC: alpha_PII_, J_PII_, P_max_, σ_PII_, F_v_/F_m_ and F_o_. The following definitions are repeated from the instrument manual [37] and the terminology of these photosynthetic parameters is derived from [26,37]. alpha_PII_ defines the initial rate at which photons are used to drive PSII photochemistry during a single turnover (ST) pulse. This parameter is a proxy of saturation of PSII photochemistry. A value of alpha_PII_ between 0.042 and 0.064 is considered optimal and reflects a good ST curve fit. J_PII_ measures the photon flux through the absorption cross section for PSII photochemistry provided by a single PSII complex. J_PII_ is interpreted as electron transport rate (ETR) on the assumption that each photon used to drive PSII photochemistry results in the transfer of an electron out of PSII. ETR, in turn, determines the rate of electron transport which controls phytoplankton carbon fixation and growth [42]. ETR can express the ability of the phytoplankton to achieve metabolic processes. P_max_ is the maximum specific photosynthetic rate which expresses the phytoplankton photosynthetic capacity in optimal ambient light conditions. σ_PII_ is the absorption cross section of PSII, which is the product of the absorption cross section for PSII light-harvesting and the probability that an absorbed photon will be used to drive PSII photochemistry. F_v_/F_m_ measures the photochemistry efficiency, with F_v_ the variable part of fluorescence obtained from the difference between F_o_ and F_m_, the minimum and maximum fluorescence, respectively. Values of F_o_, F_v_/F_m_, alpha_PII_ and σ_PII_ tend to decrease along the light steps of each FLC. The largest value of these parameters may be observed at or shortly after the first light step. Consequently, reported values of F_o_, F_v_/F_m_, alpha_PII_ and σ_PII_ were averaged over the first five light steps to characterise each FLC. The maximum value in each FLC was selected to describe the ETR parameter. P_max_ is provided once per FLC.

ST curves were recorded at both emission wavebands (685 and 730 nm) and with the B and GOR protocols, prior to running each FLC. Excitation spectra of F_v_(λex, 685) and σ_PII_(λex, 685) were also collected before running FLCs. Depending on the F_o_(452, 685) response as a proxy for the presence of chlorophylls *b* and *c*, the LabSTAF optimises and scales the excitation spectrum to the response from either blue or orange excitation [37], here referred to as algae and cyanobacteria-optimised protocols, respectively.

### 2.3. Microscopy

Microscopy counts were collected from samples fixed in Lugol’s medium. These samples were placed in a 1 mL Sedgewick Rafter Counting Chamber etched with a 20 row × 50 column grid. Samples were examined with a LEICA DM IRB inverted microscope. 100 units of 1μL each were randomly selected and counted to phytoplankton group level to quantify the abundance of diatoms, cyanophytes, chrysophytes, chlorophytes, euglenoids, dinoflagellates and unspecified single-celled eukaryotes. Counts were then multiplied by 10 to yield the abundance per mL.

Microscopy counts and identification to species level were also available from the water utility company (South West Water), from April 2002 to present, generally at monthly intervals. These long-term monitoring data were only used in Figure 2 to inform planning of the sampling campaign. Identification was made to species level, whereas the results reported here are aggregated to either “eukaryotic algae” or “prokaryotic cyanobacteria”. The samples were collected from water pumped at the draw-off tower from approximately 5 or 10 m depth, depending on surface water level.

### 2.4. Nutrients

Dissolved nutrient concentrations including nitrate + nitrite, nitrite, phosphate, ammonium and silicate were determined from 0.2-µm filtrate (Nalgene™ Sterile Syringe Filters 0.2 µm surfactant-free cellulose acetate membrane) using a 5-channel segmented flow colorimetric SEAL Analytical AAIII autoanalyser. The analytical methods were as described in [43].

## 3. Results

### 3.1. Phytoplankton and Nutrient Dynamics

**Figure 3:**
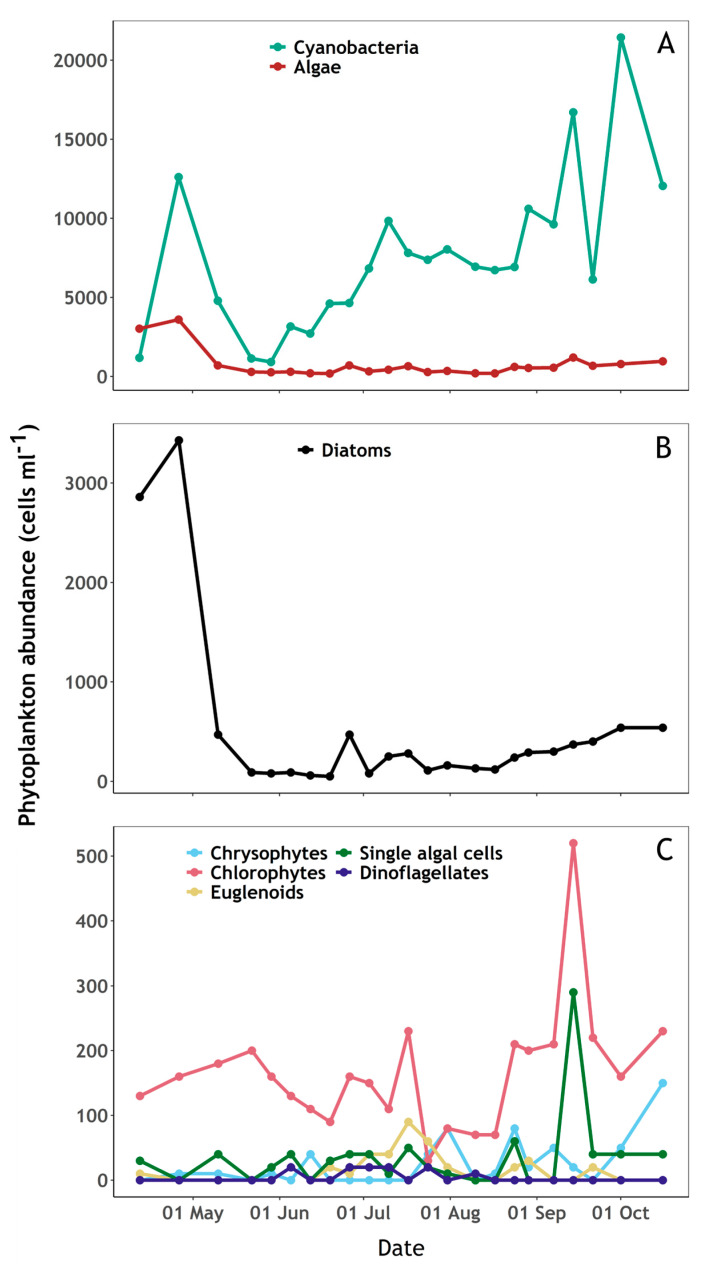
Phytoplankton abundance (cells mL^−1^) during the sampling period at Roadford Lake. (**A**): Cyanobacteria and algae. (**B**): Diatoms. (**C**): Chrysophytes, Chlorophytes, Euglenoids, Dinoflagellates and single algal cells.

**Figure 4:**
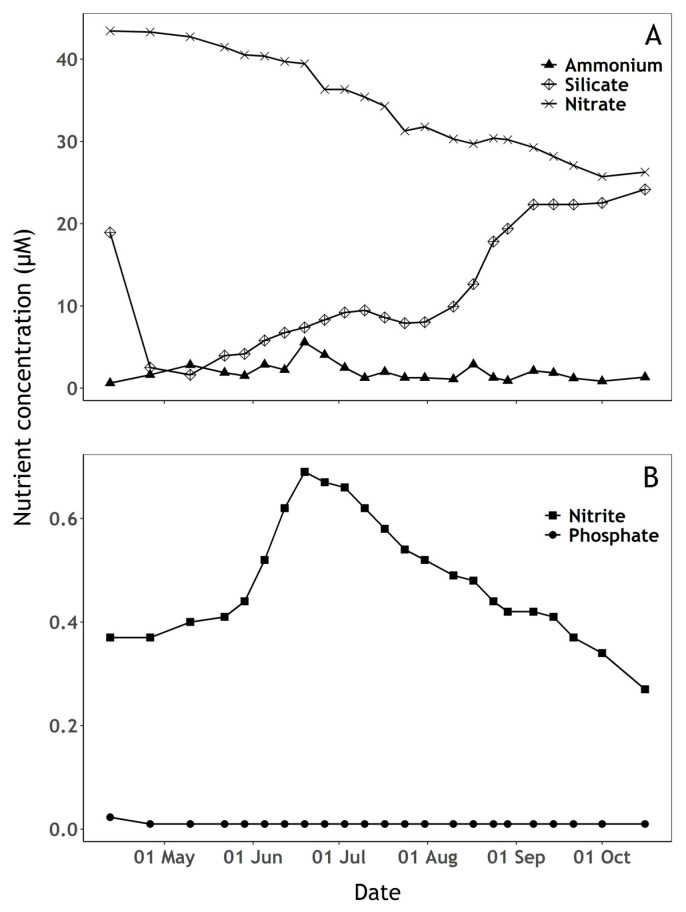
Nutrient concentrations (µM) over the sampling period. (**A**) Ammonium, silicate and nitrate concentration, (**B**) Nitrite and phosphate concentration. When the detection limit was not passed, phosphate concentrations below the limit of detection (0.02 µM) are displayed as 0.01 µM.

Algae were dominant on the first sampling day followed by cyanobacterial dominance during the rest of the campaign (Figure 3A). Diatoms and chlorophytes dominated the algal community, with diatoms varying over a wide range between samples (Figure 3B,C). A succession of phytoplankton groups was observed through the sampling period and followed variability in nutrient concentrations (Figure 3 and Figure 4). The beginning of the sampling period was marked by an initial increase in diatoms, cyanobacteria and chlorophytes until late April, during which silicate and phosphate concentrations decreased whilst a slight increase of available ammonium was observed. Cyanobacteria and diatoms abundance then decreased sharply until late May, while chlorophytes continued to increase, nitrate decreased, and nitrite availability slowly increased. From mid-May to mid-August, cyanobacteria increased while algae abundance remained relatively low. During this period, a decrease in nitrate and increase in nitrite was observed, while silicate and ammonium availability peaked. From mid-August until the end of the sampling period, algae abundance (notably diatoms and chlorophytes) increased while cyanobacteria showed an overall increasing trend with high variability between samples. Meanwhile, a slow increase of silicate and a decrease of nitrate and nitrite was observed.

**Figure 5:**
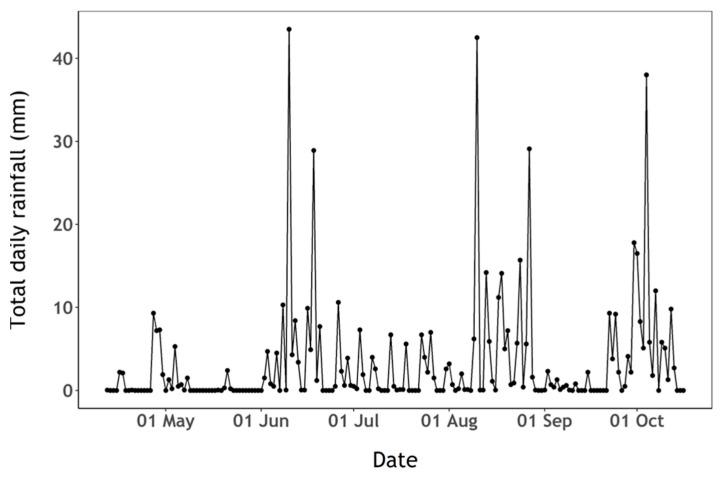
Total daily rainfall (mm) observed at the Virginstow, Beaworthy weather station.

There was evidence of phosphate limitation during the whole sampling period (Figure 4B). Phosphate concentration was slightly above the detection limit on the first sampling date and below the limit of detection (0.02 µM) during the remaining period (Figure 4B). Ammonium concentration (Figure 4B) peaked in mid-July, following a period of high precipitation during that period (Figure 5) which likely replenished both nitrite and ammonium from surrounding agricultural sources (Figure 4A,B). Silicate availability is a common driver of early-season phytoplankton succession. Silicate concentration decreased at the beginning of the sampling period when diatoms were relatively abundant, then increased up to the end of the sampling period while cyanobacteria were dominant and available ammonium appeared to be rapidly taken up (Figure 3 and Figure 4A).

### 3.2. Fluorescence Dynamics

**Figure 6:**
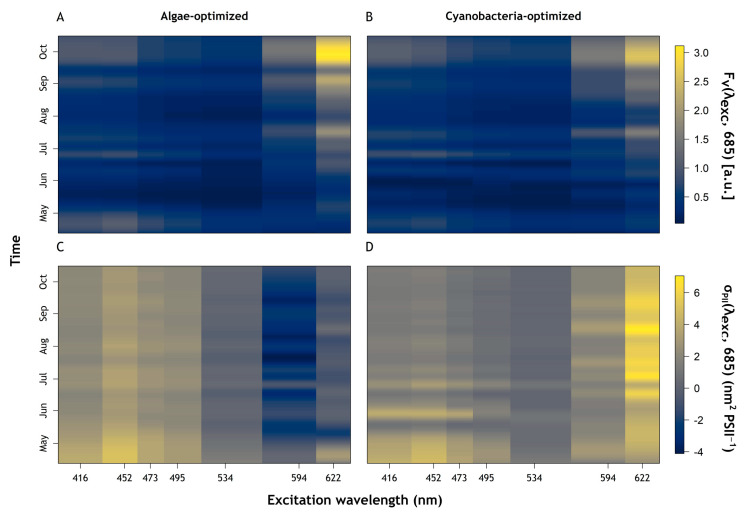
Photosynthetic Excitation spectrum results. (**A**) F_v_(λ_exc_, 685) [a.u.], algae-optimised. (**B**) F_v_(λ_exc_, 685) [a.u.], cyanobacteria-optimised. (**C**) σ_PII_(λ_exc_, 685) (nm^2^ PSII^−1^), algae-optimised. (**D**) σ_PII_(λ_exc_, 685) (nm^2^ PSII^−1^), cyanobacteria-optimised.

The succession towards increasing cyanobacteria abundance and dominance was clearly reflected in F_v_(λ_ex_, 685) and σ_PII_(λ_ex_, 685) (Figure 6). F_v_(λ_ex_, 685) showed similar dynamics between the algae and cyanobacteria-optimised protocols (Figure 6A and Figure 6B, respectively). F_v_(594, 685) and F_v_(622, 685) increased throughout the sampling period, matching changes in cyanobacteria abundance (see Figure 3A) while higher values of F_v_(416, 685) and F_v_(452, 685) corresponded to higher abundance of algae at the beginning of the sampling period (Figure 6A,B). It should be noted that the algae-optimised protocol showed higher values of F_v_ than the cyanobacteria-optimised protocol (Figure 6A,B). σ_PII_(λ_ex_, 685) showed distinct responses from different parts of the community between the two protocols (Figure 6C,D). The blue part of the spectrum showed a gentle decrease in σ_PII_ under the algae-optimised protocol while under the cyanobacteria-optimised protocol, σ_PII_ was higher in early April with strong short-term variations until Mid-July. These variations showed contrasting behaviour between blue to orange–red excitation wavebands for σ_PII_ in the cyanobacteria-optimised protocol (Figure 6D), largely absent in the algae-optimised result (Figure 6C). Differences in σ_PII_ between the algae and cyanobacteria-optimised protocols were most significant in the red–orange excitation wavebands. σ_PII_(622, 685) increased markedly in the middle of the sampling period using the cyanobacteria-optimised protocol (Figure 6D) while σ_PII_(534, 685), σ_PII_(594, 685) and σ_PII_(622, 685) showed lower values under the algae-optimised protocol.

### 3.3. Relative Cyanobacteria Abundance and F_o_ Dynamics

**Figure 7:**
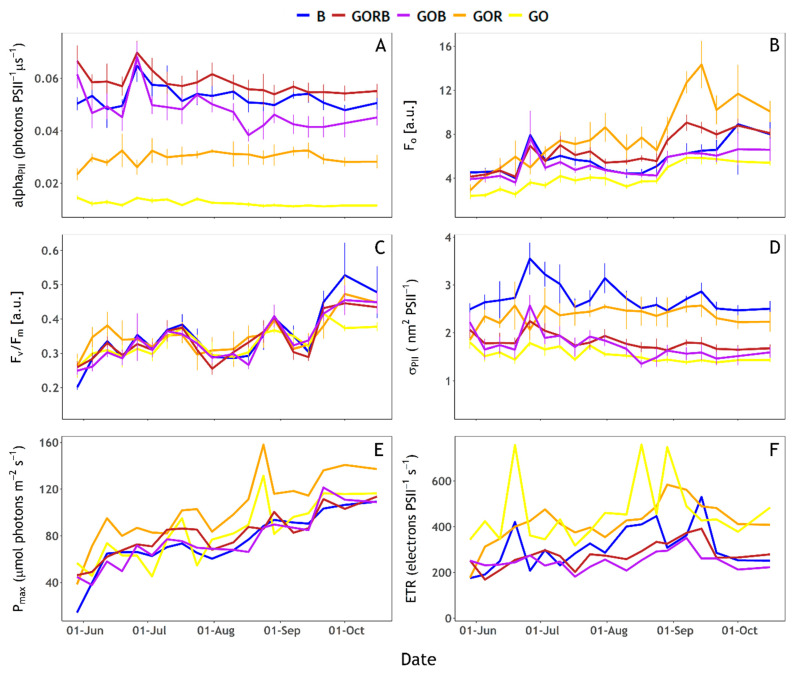
**Fluorescence Light Curves** (FLCs) photosynthetic parameters obtained with the five excitation protocols (B), GORB, GOB, GOR and GO, see Table 1). (**A**) alpha_PII_ (photons PSII^−1^ µs^−1^), (**B**) F_o_ [a.u.], (**C**) F_v_/F_m_ [a.u.], (**D**) σ_PII_ (nm^2^ PSII^−1^), (**E**) P_max_ (µmol photons m^−2^ s^−1^) and (**F**) ETR (electrons PSII^−1^ s^−1^).

F_o_(GOR) trends followed variations in cyanobacteria abundance over time (Figure 7B, and see Figure 3A). F_o_(GO) showed the same trend but with much lower values compared to F_o_(GOR), indicating that F_o_(GO) was sensitive to cyanobacteria abundance despite lower excitation energy. Abundances of algae and cyanobacteria were significantly correlated with F_o_ obtained from the five excitation protocols. The abundance of cyanobacteria was more significantly correlated with F_o_(GOR) (linear regression, *p*-value < 0.0001, adjusted R^2^ = 0.66) than with other protocols (linear regression, *p*-values between *p*-value < 0.01 (GOB) and *p*-value < 0.0001 (GORB), adjusted R^2^ ranged between 0.30 (GOB) and 0.58 (GORB)). Reciprocally, the abundance of algae was most strongly correlated with F_o_(GORB) (linear regression, *p*-value < 0.0001, adjusted R^2^ = 0.57) than other protocols (linear regression, *p*-value between *p*-value < 0.001 (GOR) and *p*-value < 0.001 (B), adjusted R^2^ ranged between 0.46 (GOR) and 0.57 (B)).

### 3.4. Photophysiological Characterisation

When targeting cyanobacteria, full saturation of PSII was not reached with the GOR and GO protocols. alpha_PII_(GO) was < 0.02 and alpha_PII_(GOR) around 0.03 (Figure 7A). The other protocols (B, GORB and GOB) did achieve saturation of the community with alpha_PII_ near or above 0.04. The fitting of ST curves to obtain photophysiological parameters does not necessarily require full saturation as long as the initial fluorescence rise and asymptote (F_m_) can be extrapolated from the curve. Nevertheless, GO protocol results should be interpreted with caution.

Photophysiological parameters derived from the FLCs showed multiple trends. P_max_, F_v_/F_m_ and F_o_ all increased over time (Figure 7B,C,E). F_v_/F_m_ increased from 0.2–0.3 at the end of May to approximately 0.4–0.5 at the end of the sampling period with minor variations between excitation protocols, detailed below (Figure 7C). By contrast, σ_PII_ showed more short-term variations and a generally decreasing trend (Figure 7D). ETR generally increased up to mid-September before it decreased again, while ETR(GO) results were highly variable (Figure 7F). Assessing specific differences between excitation protocols, P_max_(GOR) and ETR(GO) showed higher values than other protocols (Figure 7 E,F). P_max_(GOR) and P_max_(GO), as well as ETR(GOR) and ETR(GO), followed similar rising trends. When combined with B excitation (GOB and GORB protocols), these trends remained similar, whilst P_max_(B) lacked some of the short-term variability over the duration of the rising trend. ETR(B) showed a contrasting trend from ETR(GORB) and ETR(GOB). While ETR(GO) and ETR(GOR) trends seem mostly driven by cyanobacteria abundance, ETR(B) trends seem to follow algae abundance (Figure 7F). Moreover, changes in diatoms, chlorophytes and other single-celled algae abundance followed short-term variations of ETR(B) even when algal abundance was low (Figure 7F). σ_PII_(B) and σ_PII_(GOR) were both higher than the other excitation light compositions (Figure 7D). σ_PII_(B) showed three peaks in the time series during late June, late July and mid-September. Only the steady decrease following the September peak was also visible in σ_PII_(GOR). Short-term variations of σ_PII_(B) and σ_PII_(GOR) seemed to follow short-term variations of, respectively algae and cyanobacteria abundance (Figure 7D). The other protocols showed similar trends between them with values decreasing slowly from the end of May up to the end of the sampling period, and a σ_PII_ peak observed at the end of June with GORB, GOB, and GO protocols.

Photochemistry efficiency (F_v_/F_m_) was similar between excitation light protocols except marked differences at the start of June (higher F_v_/F_m_(GOR)) and at the end of September (higher F_v_/F_m_(B) and lower F_v_/F_m_(GO), Figure 7C). F_o_(GOR) was higher than F_o_ obtained with the other protocols (Figure 7B). F_o_(GO) was consistently lower compared to other combinations of excitation wavebands. F_o_(GORB) showed higher values than F_o_(B), F_o_(GOB) and F_o_(GO) protocols from July until the end of the sampling period. F_o_(GORB) was lower than F_o_(GOR), contrary to expectations considering the GORB protocol operated the GOR LEDs at the same intensities between protocols and received additional energy from the B LEDs.

### 3.5. Emission Ratio of 730 nm over 685 nm

**Figure 8:**
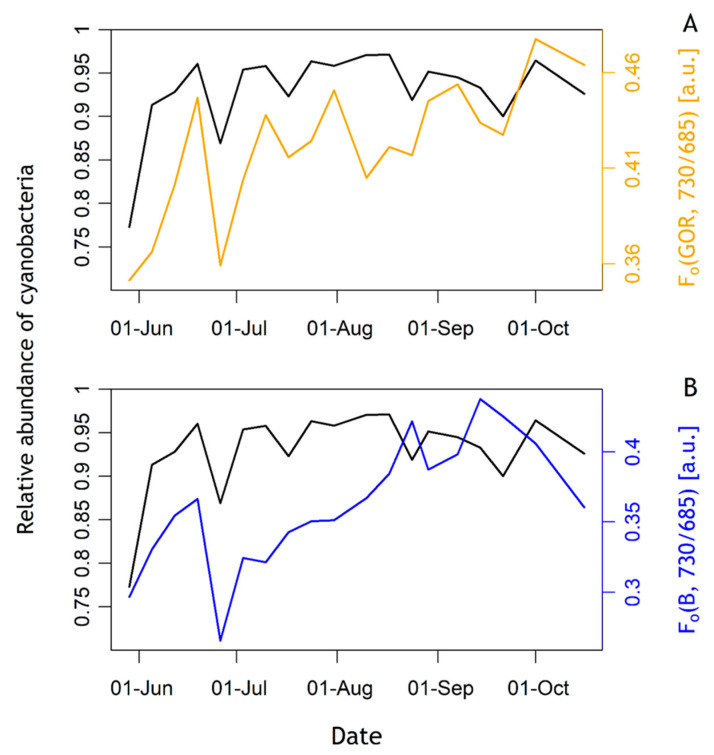
F_o_ emission ratio using B and GOR excitation protocols compared to the relative abundance of cyanobacteria (black drawn line). (**A**) F_o_(GOR, 730/685) [a.u.], (**B**) F_o_(B, 730/685) [a.u.].

The emission ratio of 730 over 685 nm, or F_o_(GOR, 730/685), corresponded to cyanobacteria biomass as seen in Figure 8. F_o_(GOR, 730/685) was positively and significantly correlated with the relative abundance of cyanobacteria of the phytoplankton community (linear regression, *p*-value < 0.01, adjusted R^2^ = 0.42). F_o_(B, 730/685), in contrast, was not significantly correlated with the relative abundance of either group (linear regression, *p*-value: 0.076, adjusted R^2^ = 0.12), although some correspondence of F_o_(B, 730/685) with cyanobacteria biomass could be observed.

## 4. Discussion

### 4.1. Phytoplankton, Nutrient and Fluorescence Dynamics

The succession of phytoplankton observed from early spring to late summer is typical of the seasonal succession of phytoplankton in temperate lakes [44,45]. The increase of cyanobacteria may be explained by a combination of several factors: temperature increase, replenishment of ammonium, and the ability of cyanobacteria to store phosphorus [46].

The use of fluorescence excitation spectra to distinguish pigments groups in phytoplankton forms the basis of all available multispectral phytoplankton fluorescence excitation instruments, and has been described in great detail e.g., [15,47]. In this study, the shift observed from algae to cyanobacteria dominance was clearly visible in F_o_(GOR), and F_o_(GO) which tracked variations of cyanobacteria abundance over time. In addition, spectra of F_v_(λ_ex_, 685) and σ_PII_(λ_ex_, 685) provide insight into the photosynthetic light uptake potential of the two major phytoplankton groups. In spectra of F_v_(λ_ex_, 685), a shift occurred from shorter to longer excitation wavebands as the relative abundance of cyanobacteria increased. Similarly, spectra of σ_PII_(λ_ex_, 685) showed distinct responses from algae and cyanobacteria. This was observed with σ_PII_(λ_ex_, 685) obtained with the algae-optimised protocol at the blue part of the spectrum, following algae abundance. On the other hand, at red–orange wavebands, higher σ_PII_(λ_ex_, 685) values obtained with the cyanobacteria-optimised protocol tracked the abundance of cyanobacteria. These results illustrate the use of PSII excitation spectra to determine the photosynthetic response by either phytoplankton group during natural succession. The fact that the algae-optimised protocol showed low σ_PII_(λ_ex_, 685) in the red–orange part of the spectrum when algae were most abundant, while the cyanobacteria-optimised protocol induced higher σ_PII_(λ_ex_, 685) at blue wavebands illustrates that both protocols induced signal in the other phytoplankton group. The former is explained by the presence of red light at 622 nm in the cyanobacteria-optimised protocol which will induce some chla absorption in both groups, while the latter points to blue light absorption by chla in algae and cyanobacterial PSII chla. The parallel use of the two protocols ensures that there is sufficient signal to extrapolate the photosynthetic parameters, and that differences are associated with the phytoplankton group for which each protocol is optimised. During periods where neither group dominates, no major differences should be expected between the protocols.

### 4.2. Emission Ratio of 730 nm over 685 nm

The F_o_ emission ratio F_o_(GOR, 730/685) was positively and significantly correlated with the relative abundance of cyanobacteria in the community. F_o_(B, 730/685) was not significantly correlated but followed the overall trend and short-term variations of the relative abundance of cyanobacteria. As discussed above, the B and GOR excitation signals are not exclusively diagnostic to either group. It is, therefore, interesting to observe that the emission ratio is sensitive to cyanobacteria in either excitation protocol. These results confirm the potential of interpretating the ratio of 730 nm over 685 nm emission to identify cyanobacteria in the community suggested by [35], as well as the importance of the choice of the excitation wavebands. LEDs targeting the orange-to-red spectrum will favour PSII emission from cyanobacteria compared to algae, whilst avoiding the excitation of chla associated with PSI further enhances the fluorescence emission ratio, as indeed observed here. Nevertheless, we did not observe major variability in time in the relative abundance of cyanobacteria in the community, so these results should be interpreted with some caution. To further confirm the utility of F_o_(GOR, 730/685) to identify cyanobacteria, measurements should be repeated alongside group-specific biomass determinations at higher frequency. In this context, the light-acclimation and nutrient history of the samples would have to be considered as these can be expected to influence the ratio of PSI over PSII emission through the expression of accessory pigment as well as photophysiological efficiency. It is known that state transitions induce further variations of the PSII to PSI ratio [48]. In this study, samples were dark-adapted before measuring F_o_ at both emission wavebands so that state transitions should not influence the observed variability in F_o_(GOR, 730/685). Moreover, as described by [49], light conditions in which the cyanobacterium *Synechocystis sp*. PCC 6803 were grown as well as the excitation wavebands have an influence on the PSII:PSI ratio. Ref. [49] showed that the cyanobacterium grown under blue light had a lower PSI:PSII ratio than under orange or red light. Under blue excitation (440 nm), PSI:PSII ratios were higher than under orange excitation (590 nm) [49]. This phenomenon is explained by the fact that the phycobilisomes in cyanobacteria do not efficiently transfer energy from blue light absorption to PSII.

### 4.3. Photophysiological Characterisation

Differentiated trends between the photosynthetic parameters highlight changes in the physiology of phytoplankton in the lake. The photochemistry efficiency expressed by F_v_/F_m_ more than doubled over the observed period, likely corresponding with improved nutrient availability to the dominant group. F_v_/F_m_ did not vary between the protocols, suggesting that algae and cyanobacteria populations adjusted similarly to environmental conditions (as also observed by [22]). In the case of the algal population, this included replacement of diatoms by chlorophytes during a period of silicate depletion.

P_max_ and F_o_ expectedly increased with overall biomass. σ_PII_ showed a decreasing trend over the sampling period which could be associated with seasonally increasing light availability. Ref. [50] also showed an inverse correlation between F_v_/F_m_(478, λ_em_) and σ_PII_(478, λ_em_), which can be explained by energy requirements: the increasing numbers of pigment molecules in the light-harvesting antenna induce a higher probability of thermal dissipation and a decrease of photochemistry efficiency [50,51]. Moreover, the excitation spectra of σ_PII_ showed higher values of σ_PII_(560, λ_em_) than σ_PII_(478, λ_em_) in cyanobacteria while the opposite was observed in algae [50]. In our study, algae abundances were lower than cyanobacteria except at the beginning of the sampling period. We would, thus, expect to see higher σ_PII_(GOR) than σ_PII_(B) during periods of cyanobacteria dominance. Nevertheless, σ_PII_(B) and σ_PII_(GOR) followed the short-term variations in algae and cyanobacteria abundance, respectively. The fact that σ_PII_(B) was higher than σ_PII_(GOR) can be explained, either by the fact that σ_PII_(B) also targets cyanobacteria or that the ratio of chla pigments over phycobilipigments was low. This corroborates with the fact that phytoplankton growth was not light-limited and that cyanobacteria regulate their pigment expression [9]. Moreover, σ_PII_ is calculated as the product of alpha_PII_ and EST which is the photon irradiance provided to the sample by the LEDs during a ST pulse (σ_PII_ = alpha_PII_ × 100 EST, see [37]). Our results suggest lower alpha_PII_ and EST values induced by the GOR protocol compared to the B protocol, which can explain the lower values of σ_PII_(GOR) compared to σ_PII_(B). The interpretation of σ_PII_ obtained with the GOR protocol and the B protocol is, therefore, not straightforward here.

PAM (Pulse Amplitude Modulation) and FFR (Fast Repetition Rate) Fluorometers using several induction protocols have been used to estimate the physiology and the biomass of distinct phytoplanktonic groups, including cyanobacteria. Ref. [23] used the FastOcean (FRRf) and a combination of LEDs to accurately estimate ETR_PSII_ of freshwater cyanobacteria. Ref. [52] could discriminate cyanobacteria from other phytoplanktonic groups using a PAM fluorometer and blue, green and red LEDs. Ref. [53] warned that ST fluorometers equipped solely with blue LEDs lack sensitivity to cyanobacteria without phycoerythrin. Some improvements to group-specific and bulk phytoplankton sensitivity are therefore expected from modern instruments such as used here, equipped with multiple excitation channels and broad-spectrum actinic light. In this work, P_max_ increased over the sampling period and was higher when obtained with GOR and GO protocols, explained by the increased abundance of cyanobacteria over time. This confirms the importance of using protocols targeting cyanobacteria and especially the GOR protocol (or the GO protocol if saturation can be improved) when trying to predict group-specific population growth. Short-term variations of P_max_ obtained with GOR and GO protocols followed cyanobacteria abundance over time while short-term variations in P_max_ obtained using the other excitation protocols followed algae abundance. The general increasing trend of P_max_ between the excitation protocols suggest that protocols other than GOR and GO also induced a signal in cyanobacteria. ETR generally increased up to mid-September and subsequently decreased with ETR(GOR) and ETR(GO) showing higher values than other protocols. ETR(GOR) followed trends driven by cyanobacteria abundance while ETR(B) trends followed abundance of algae. Moreover, ETR(B) showed a different trend from P_max_, especially at the end of the sampling period. Wide variability in the ‘electron-to-carbon exchange rate’ was described by [54], which highlights the difficulty in linking ETR to C fixation. This is confirmed by the fact that this study focuses on a single lake over a relatively short period of time while nutrient concentrations and light availability remained relatively stable. It was explained by [55] that alternative electron sinks can occur for other metabolic processes and physiological mechanisms. Moreover, light and nutrient history effects on ETR values cannot be determined well from this data set due to the relatively long sampling intervals.

### 4.4. Suitability of LabSTAF for In Situ Assessment of Cyanobacteria

The configuration of the LabSTAF used in this study could not guarantee PSII saturation in the protocols we expected to be most diagnostically sensitive to cyanobacteria, which should be improved using higher intensity LEDs. Ideally, the R LED would only be used comparatively (i.e., combined with B or GO), to determine how well the GO protocol describes the cyanobacteria component of the community. A complete assessment would consist of the spectral excitation measurements providing insights into the efficiency of light uptake through key pigments in the community (providing the first insight into cyanobacteria presence) followed by GO and B fluorescence–excitation curves to determine photophysiological growth parameters which would indicate whether any differences in photosynthetic efficiency are likely between the two major phytoplankton groups.

Due to the nature of overlapping photosynthetic pigment absorption profiles between the major phytoplankton groups, it is essential to consider several optical markers, as confirmed by results presented here. Notably, we recommend using in parallel the ratio of F_o_(GOR, 730/685) and the PSII excitation protocols obtained with cyanobacteria and algae-optimised protocol. Ultimately, parameters derived from the FLCs obtained with GOR and B protocols can then be used to gain insight into likely growth and succession in the phytoplankton community. The DWM emission feature is promising because replacement filters could, in theory, be considered to look at other diagnostic wavebands for targeting cyanobacteria using this approach. The 650 nm emission waveband has been recommended by [35] to be implemented in a fluorometer targeting cyanobacteria. Although emission recorded at 650 nm could yield weak fluorescence compared to emission recorded at 685 nm, F_o_(GOR, 650/685) would likely give additional valuable diagnostic information on the presence of phycobilisome pigments.

## 5. Conclusions

This study assessed the ability of a new active fluorometer, the LabSTAF, to diagnostically assess the physiology of freshwater cyanobacteria in a reservoir exhibiting annual blooms.

The reservoir had a typical seasonal succession of phytoplankton of a temperate lake with diatoms being more abundant in spring and cyanobacteria more abundant in summer. PSII excitation spectra optimised for algae or for cyanobacteria showed distinct responses, illustrating the suitability of the instrument to determine the photosynthetic response by either group during their natural succession. FLCs parameters obtained with GOR and B protocols captured physiology parameters of, respectively, cyanobacteria and algae. However, the GO protocol, expected to be most diagnostically sensitive of cyanobacteria, did not reach saturation. Moreover, F_o_(GOR, 730/685) was significantly correlated with the relative abundance of cyanobacteria, which shows the potential of the ratio of 730 nm over 685 nm emission to identify cyanobacteria in a mixed phytoplankton community.

Due to the nature of overlapping photosynthetic pigment absorption profiles between the major phytoplankton groups, it is essential to consider several optical markers. We recommend using in parallel excitation spectra obtained with both protocols and F_o_(GOR, 730/685) to detect different pigment groups and assess cyanobacteria presence, followed by FLCs obtained with GOR and B protocols to assess the physiology and potential to grow of cyanobacteria and algae. Increased intensity of GO LEDs should be achieved to correctly assess the physiology of cyanobacteria and the R LED should only be used comparatively. Moreover, a 650 nm bandpass filter could be implemented in the DWM feature of the LabSTAF to measure the ratio of 650 nm over 685 nm emission, F_o_(GOR, 650/685). The potential of F_o_(GOR, 650/685) to be diagnostic of cyanobacteria should also be verified. According to this study, the LabSTAF is a good candidate to assess the presence and physiology of cyanobacteria in the natural environment.

This instrument is well suited for long-term continuous measurements and its optical features provided suitable optical markers to target cyanobacteria. Improvement or addition of some features are nevertheless recommended to increase the potential of the LabSTAF to be diagnostic of cyanobacteria.

## Figures and Tables

**Table 1:** Excitation protocols tested to target pigments associated with specific phytoplankton groups, as described by [40,41]. Phycobilipigments include phycoerythrin, allophycocyanin and phycocyanin.

Protocol	Excitation Wavebands (nm at Centre)	Intensity Range (μmol Photons m^−2^ s^−1^ )	Pulse Length (μs)	Photosynthetic Pigment Groups Targeted	Phytoplankton Group Targeted
B	452, 452	28,175–33,538	100	Chlorophylls *a*/*b*/*c*, carotenoids	Algae, weak signal from cyanobacteria possible
GOR	534, 594, 622	20,990	200	Phycobilipigments Chlorophylls *a*/*b*/*c* and carotenoids	Cyanobacteria with weaker signal from algae likely
GORB	452, 452, 534, 594, 622	49,513–54,528	200	Chlorophylls *a*/*b*/*c*, carotenoids and phycobilipigments	Whole community
GOB	452, 452, 534, 594	41,919–46,933	200	Chlorophylls *a*/*b*/*c*, carotenoids and phycobilipigments	Whole community with a slower saturation response compared to GORB
GO	534, 594	13,395	200	Phycobilipigments, Chlorophyll *c*, carotenoids	Cyanobacteria, cryptophytes and rhodophytes

## Data Availability

The data underlying this paper are held in a public repository under digital object identifier 10.5281/zenodo.7469162.
